# Aerosols enhance cloud lifetime and brightness along the stratus-to-cumulus transition

**DOI:** 10.1073/pnas.1921231117

**Published:** 2020-07-13

**Authors:** Matthew W. Christensen, William K. Jones, Philip Stier

**Affiliations:** ^a^Atmospheric, Oceanic and Planetary Physics, University of Oxford, Oxford OX1 3PU, United Kingdom

**Keywords:** aerosols, clouds, radiative forcing

## Abstract

Anthropogenic aerosols are hypothesized to enhance planetary albedo and offset some of the warming due to the buildup of greenhouse gases in Earth’s atmosphere. Aerosols can enhance the coverage, reflectance, and lifetime of warm low-level clouds. However, the relationship between cloud lifetime and aerosol concentration has been challenging to measure from polar orbiting satellites. We estimate two timescales relating to the formation and persistence of low-level clouds over $1^{○}\times 1^{○}$ spatial domains using multiple years of geostationary satellite observations provided by the Clouds and Earth’s Radiant Energy System (CERES) Synoptic (SYN) product. Lagrangian trajectories spanning several days along the classic stratus-to-cumulus transition zone are stratified by aerosol optical depth and meteorology. Clouds forming in relatively polluted trajectories tend to have lighter precipitation rates, longer average lifetime, and higher cloud albedo and cloud fraction compared with unpolluted trajectories. While liquid water path differences are found to be negligible, we find direct evidence of increased planetary albedo primarily through increased drop concentration ($N_{d}$) and cloud fraction, with the caveat that the aerosol influence on cloud fraction is positive only for stable atmospheric conditions. While the increase in cloud fraction can be large typically in the beginning of trajectories, the Twomey effect accounts for the bulk (roughly 3/4) of the total aerosol indirect radiative forcing estimate.

## Significance

All cloud droplets on Earth form from tiny airborne particles known as aerosols. Additional aerosols from anthropogenic activity have produced more cloud droplets but at smaller sizes. The smaller, more numerous droplets in clouds do not collide as effectively, therefore resulting in less precipitation. Using a combination of time-lapse satellite imagery and air mass trajectory modeling, we show that aerosols can enhance cloud fraction and extend the lifetime of overcast cloud fields primarily under stable atmospheric conditions as typically found off the west coasts of subtropical stratocumulus-dominated regions. Longer-lived clouds have a stronger cooling influence on climate and therefore, need to be correctly parameterized in atmospheric models so that accurate projections of climate change can be achieved.

The planetary albedo and energy budget of the Earth is strongly regulated by clouds. An increase in anthropogenic aerosol typically increases cloud condensation nuclei and cloud droplet number concentration ($N_{d}$). As $N_{d}$ increases, the Twomey effect predicts an increase in cloud albedo and decrease in cloud droplet size for the specific condition that the amount of condensed water remains constant (1). Smaller droplets in polluted clouds can result in reduced collision coalescence and delay the onset of precipitation and accumulation of liquid water path. Over time, these responses can result in the expansion of cloud horizontal and vertical extents (2). By contrast, liquid water path and cloud albedo can also decrease under some conditions for clouds with larger concentrations of small droplets [for example, in ship, volcano, and industrial pollution tracks (3)] owing to an enhanced entrainment–evaporation aerosol feedback in dry atmospheric conditions (4). The net effect of these processes has been hypothesized to influence the lifetime of clouds (5).

The existence of an aerosol effect on cloud lifetime is widely disputed (6, 7). The dispute partly arises from the lack of a clear definition and measurements that are resolved over the dimension of time. The forcing can be decomposed into liquid water path and cloud fraction adjustments, but these do not necessarily act in unison (8) to produce larger radiative effects and hence, longer cloud lifetimes. A common approach to quantifying the aerosol effect on cloud lifetime is to use statistical relationships in cloud micro- or macrophysical properties (e.g., precipitation rate, cloud liquid water content, or cloud fraction changes) to changes in aerosol concentration. However, typically these satellite-derived relationships are computed using a static Eulerian framework, which does not permit the analysis of cloud fields evolving through time. The A-Train constellation has been paramount for advancing process-level understanding of clouds and aerosols from its unique multisensor array (radiometers, a lidar, and a cloud-profiling radar), but short-term temporal changes cannot be quantified from a sun-synchronous orbit. The A-Train can provide only single snapshot images of cloud systems once per day at 13:30 local time in the afternoon (and at 01:30 but without optical property retrievals), thereby constraining the observations to an Eulerian framework. The connection of these variables to the dimension of time in a Lagrangian framework offers the capability to examine the aerosol influence on cloud development and lifetime. In this work, we use time-resolved observations from satellites in geostationary orbit and define cloud lifetime as the amount of time the cloud fraction computed over large spatial domains ($1^{○}\times 1^{○}$) exceeds a certain threshold (0.75).

The Lagrangian framework has traditionally been used to study the time-dependent response of the marine stratus-to-cumulus transition zone in observational (9–11) and modeling (12–14) studies. This transition typically follows a three-day equatorward redundant trajectory where the boundary layer proceeds through several diurnal cycles (13), increasingly warmer sea surface temperatures and unstable atmospheric conditions, stronger surface moisture and sensible heat fluxes, and more frequent precipitation. Sea surface temperature gradients and free-tropospheric subsidence rates are considered the primary drivers for the stratus-to-cumulus transition (13). However, changes in aerosol loading may also play a significant role in the development of clouds through their ability to modify $N_{d}$, precipitation rates, and cloud-top entrainment rates (4). Large eddy simulation experiments suggest that larger $N_{d}$ can extend the stratus-to-cumulus transition zone several hours by delaying the onset of precipitation and breakup of the stratocumulus layer (14). Furthermore, the transition can be hastened or slowed by the presence of solar-absorbing smoke layers. The response depends on a variety of factors: the distance between the top of the stratocumulus cloud and location of the overlying smoke layer, whether the smoke mixes with the planetary boundary layer (PBL), and whether the smoke is accompanied by an increase in moisture (15). A primary goal of the analysis undertaken here is to quantify the extent to which aerosols influence cloud properties along the stratus-to-cumulus transition zone using temporally resolved satellite observations.

## Lagrangian Trajectory Framework

Several operational satellite products are collocated to Lagrangian trajectories calculated using the Hybrid Single-Particle Lagrangian Integrated Trajectory (HYSPLIT) model (*Materials and Methods*). Geostationary satellite retrievals of the radiative fluxes, cloud properties, and aerosol properties are taken from the Clouds and the Earths Radiant Energy System (CERES) Edition 4a (Ed4a) Synoptic (SYN) 1 degree 1 hour (1deg1hr) product (16). Precipitation estimates are temporally resolved every half hour in the Integrated Multi-satellitE Retrievals for Global Precipitation Measurement (IMERG) V06B product through an algorithm that integrates, interpolates, and intercalibrates infrared brightness temperature data from numerous satellites in geostationary orbit and from microwave retrievals from several satellites in nongeostationary satellite orbit. Sun-synchronous cloud and aerosol retrievals are obtained from the MODerate Resolution Imaging Spectroradiometer (MODIS) collection 6.1 product. Satellite retrievals are spatially aggregated (or linearly interpolated) to a $1^{○}\times 1^{○}$ region that moves along the centerline of the calculated positions in the HYSPLIT trajectory. This domain is small enough to avoid averaging over spatial gradients in aerosol properties (17) yet large enough to encompass a wide range of spatial resolutions from each satellite product. All products are averaged over hourly intervals along Lagrangian trajectories.

**Fig. 1.**
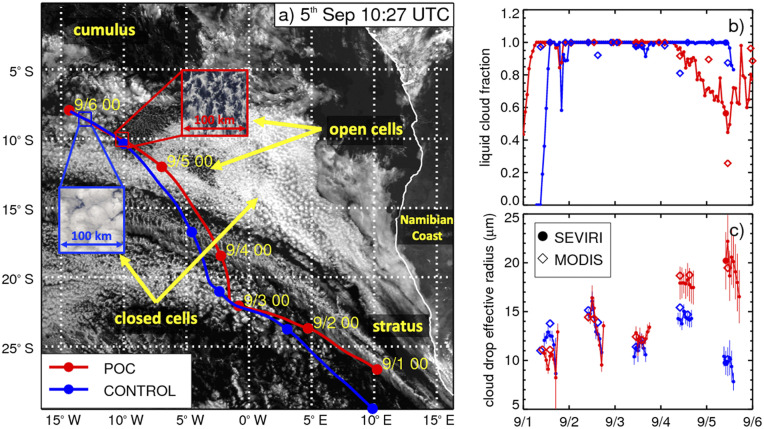
(*A*) SEVIRI 0.64-μm reflectance image obtained on 5 September 2017 at 10:27 universal time coordinated overlain with the HYSPLIT back trajectories ending inside the POC (red) and outside in the overcast clouds (blue). Time series of (*B*) cloud fraction and (*C*) cloud drop effective radius along the trajectory as retrieved from SEVIRI (circles and solid line) and MODIS (diamonds) NASA products. Large filled circles on the time series plots coincide with the satellite image. Variability is represented by one full SD (vertical lines) calculated over the spatial domain (squares shown in *A*).

Fig. 1 and *SI Appendix*, Fig. S1 demonstrate how the collocation method is implemented. The satellite image shows two Lagrangian trajectories covering the breadth of the stratus-to-cumulus transition zone. This case coincides with the emergence and dissipation of a pocket of open cells (POC) that formed on 5 September 2017 off the west coast of Namibia. POCs occasionally form along the stratus-to-cumulus transition and exhibit an open cellular hexagonal-like structure that results in relatively low planetary albedo compared with the bright surrounding deck of closed cell clouds. They typically form during the night after precipitation has become heavy enough to efficiently scavenge accumulation mode aerosol (18). Very low concentrations of accumulation mode aerosols, low $N_{d}$, and heavy precipitation rates are commonly observed in POCs. Cloud evolution is examined using a HYSPLIT back trajectory initialized from inside the location of the POC (red line) and in another trajectory located just to the east of the POC (blue) in the surrounding closed cell clouds.

The Spinning Enhanced Visible and Infrared Imager (SEVIRI)-retrieved liquid cloud fraction rapidly increases to complete saturation over the trajectories as they are advected northwesterly over a four-day period. The initial aerosol optical depth (AOD) is similar in both trajectories with a value of 0.19±0.05. The cloud fraction remains nearly at 100% until the POC begins to form on 5 September at 4 AM local time. Afterward, cloud fraction declines for roughly 24 h. The geostationary satellite observations show the unique ability to accurately determine the exact timing of the POC formation (within 1 h), while the sparse data from MODIS (Terra and Aqua satellites) only provide a rough estimate (within 12 h). Cloud droplet effective radius markedly increased over the development period of this POC (Fig. 1*C*). Similarly, the $N_{d}$ is markedly decreased inside the POC (24 ± 4 ${cm}^{-3}$) compared with the surrounding cloud field (174 ± 29 ${cm}^{-3}$) using Eq. **2** described below. Satellite retrievals are generally more uncertain in broken cloudy conditions due to the general inability to account for complex three-dimensional (3D) radiative transfer and absorption (19). Higher effective radius and rain rates were observed inside this POC during the CLouds-Aerosol-Radiation Interactions and Forcing flight campaign (20 to 40 μm) compared with the surrounding cloud deck (10 to 20 μm) as described in Abel et al. (20). Similar behavior was observed in open cellular broken cloud conditions during the Variability of the American Monsoon Systems Ocean-Cloud-Atmosphere-Land Study campaign (21) and from a climatology of POCs in satellite observations (20). We conclude that there is confidence in the ability of the satellite data to capture microphysical responses along the trajectories used in this study.

## Results

Time-resolved satellite retrievals of aerosol, cloud, and radiation are examined from several thousand 80-h forward trajectories for 10 y of observations (2008 to 2017). A key challenge to studying aerosol–cloud interactions is isolating the impact of confounding variables (e.g., meteorological variables such as sea surface temperature) on cloud properties so that the causal relationship can be quantified. Trajectories are initialized in clear-sky areas and sorted by clean and polluted conditions based on AOD (*Materials and Methods*). We use two strategies to account for confounders: 1) a regional-scale analysis of three subtropical oceanic basins in which all trajectories are constrained by wind direction to transect similar gradients in sea surface temperature and 2) a global-scale analysis where trajectories are stratified by gradients in the trajectory time series of lower troposphere stability. Finally, we determine whether aerosols can extend the average lifetime of a domain-wide population of clouds from timescale metrics applied to cloud development.

### Regional-Scale Analysis.

**Fig. 2.**
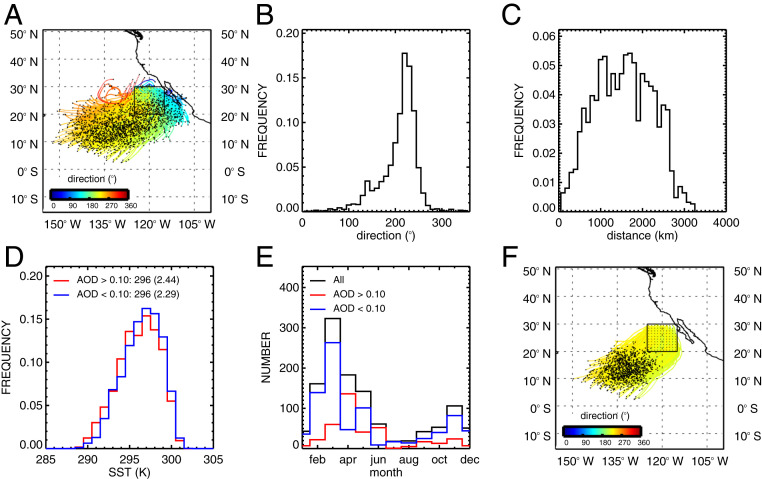
(*A*) Ensemble of forward trajectories initialized in clear-sky conditions from 2008 to 2017 off the coast of Baja California ($125^{○}$W to $120^{○}$W; $20^{○}$N to $25^{○}$N). The 80-h trajectories are calculated using HYSPLIT and GDAS meteorology. The direction (south is defined as $180^{○}$) is determined from its starting location (black square region) to the final position. Histograms of the direction (*B*) and length (*C*) are shown for the composite shown in *A*. Trajectories are constrained to flow by the median wind direction (210±10°) and cover at least 1,000 km as displayed in *F*. Histograms of ECMWF ERA-Interim sea surface temperature (SST) and the monthly occurrence for unpolluted (AOD<0.10; blue) and polluted (AOD>0.10; red) conditions are shown (*D* and *E*, respectively).

Baja Californian, Chilean, and Namibian regions are used to characterize aerosol responses along the classic stratus-to-cumulus transition zone. Over 2,000 trajectories are initialized near the coast of Baja California ($125^{○}$W to $115^{○}$W; $20^{○}$N to $30^{○}$N) (Fig. 2*A*). The majority flow in a southwesterly direction (Fig. 2*B*) covering more than 1,000 km (Fig. 2*C*). Analysis was limited to those trajectories that flow along the median wind direction (210 ± 10°) and span a distance of at least 1,000 km from their point of origin. This method ensures that all trajectories transit through similar gradients in meteorological conditions as shown by the distribution of sea surface temperature in Fig. 2*D*. The ratio of polluted to clean trajectories varies from month to month (Fig. 2*E*). However, the aerosol effect on $N_{d}$ is robust in every season, and therefore, to preserve samples we use the full distribution of months shown here. The final distribution of the filtered trajectories, which is based on consistent wind direction and distance, is shown in Fig. 2*F*. Overall, the aim of this approach is to ensure the consistency in meteorological sampling between polluted and clean trajectories, and while the procedure results in the removal of roughly one-third of the tracks that deviate from the median wind, we retain several thousand trajectories to analyze aerosol–cloud responses.

**Fig. 3.**
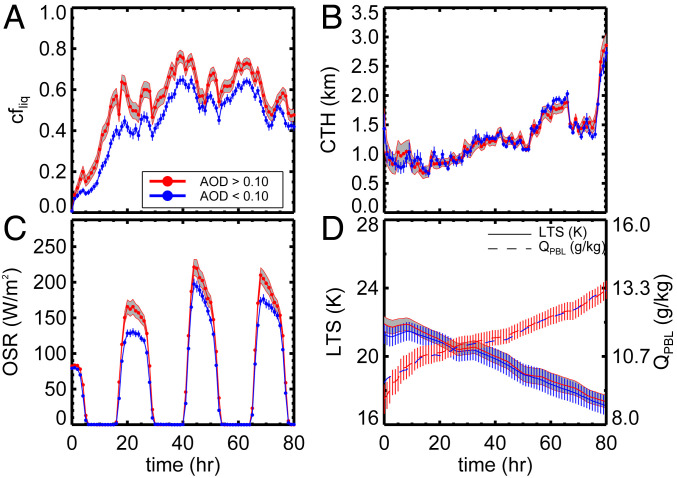
Time series of the mean (*A*) liquid cloud fraction, (*B*) CTH, (*C*) TOA outgoing shortwave radiative flux from CERES SYN, and (*D*) *LTS* and planetary boundary-layer humidity ($Q_{PBL}$) from ECMWF ERA-Interim for polluted (red lines) and unpolluted (blue lines) trajectories off the coast of Baja California. Natural variability (shaded regions and vertical bars for the unpolluted clouds) is represented by the 5th to 95th percentile CIs of the distribution.

In Fig. 3, we observe the stratus-to-cumulus transition (12) from a gradual deepening of the boundary layer as indicated by an increase in cloud top height (CTH) and atmospheric destabilization. Changes in sea surface temperature, lower troposphere stability, boundary-layer moisture, and 500-hPa subsidence rates (subsidence rates for each region shown in *SI Appendix*, Fig. S9) as a function of time are nearly identical in both clean and polluted trajectories. Two robust cloud responses are observed: 1) the diurnal cycle and 2) the influence of AOD. Cloud fraction typically increases at night through more efficient mixing of moisture throughout the boundary layer by stronger cloud top radiative cooling (12). This is displayed by the oscillatory pattern of cloud fraction as shown in Fig. 3*A*. Aerosol is also correlated to an increase in cloud fraction. Cloud fraction increases at a faster rate in polluted trajectories as indicated by the steeper slope of cloud fraction over the first 20 h of the trajectory period. While we use a strict aerosol screening criteria (*Trajectory Setup*), some influence of cloud contamination cannot be ruled out and could partially be responsible for the earlier formation of cloud at higher AOD. After this initial period of rapid cloud formation, the cloud fraction remains larger in the polluted trajectories throughout multiple diurnal cycles, albeit with smaller differences over time.

**Fig. 4.**
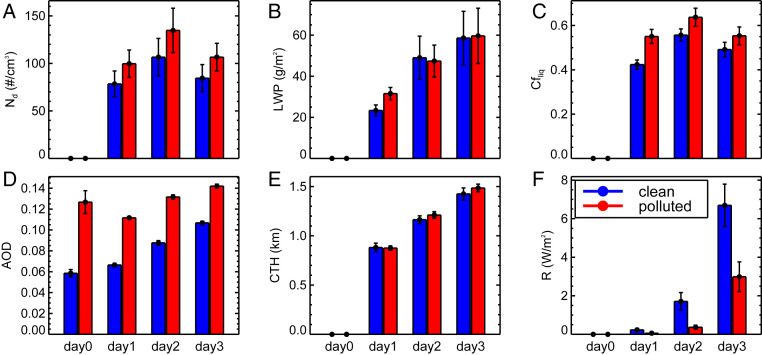
Daytime average (9 AM to 4 PM local time) (*A*) SYN $N_{d}$, (*B*) SYN liquid water path (LWP), (*C*) SYN cloud fraction ($CF_{liq}$), (*D*) CAMS AOD, (*E*) SYN CTH, and (*F*) IMERG precipitation rate multiplied by the latent heat of vaporization (i.e., R = LP, where R is the latent heat release by precipitation, L is the latent heat of vaporization and P is the surface precipitation rate) along the ensemble of trajectories off the Baja Californian coast. The 5th to 95th percentile CIs are given by the vertical bars.

Higher cloud fraction and $N_{d}$ in the trajectories forming in polluted conditions result in a significant increase in top of atmosphere (TOA) outgoing shortwave radiative flux (Fig. 3*C*). Higher cloud coverage in the polluted trajectories decreases the TOA outgoing long-wave radiative flux (by 5.2 W/$m^{2}$), but these decreases are much smaller compared with the outgoing shortwave flux (by 63.6 W/$m^{2}$). Therefore, radiative effects are based on shortwave flux changes in this paper. For deeper insight into cloud microphysical processes, we remove the nighttime portion of the diurnal cycle and focus on daily averages during daylight hours (9 to 16 local time) when the cloud optical property retrievals are most accurate from the satellite observations. $N_{d}$ and cloud fraction are significantly larger in the clouds along polluted trajectories in Fig. 4. Larger $N_{d}$ values in polluted trajectories are due to smaller cloud droplet effective radii (10% decrease) and larger cloud optical thicknesses (27.5% increase) on average. In addition, we observe some evidence for drizzle suppression in polluted clouds from the IMERG (Fig. 4*F*) and CloudSat (*SI Appendix*, Fig. S16) observations. The relatively heavier precipitation rates toward the end of day 3 may also explain the reduction in $N_{d}$ through wet deposition relative to day 2.

**Fig. 5.**
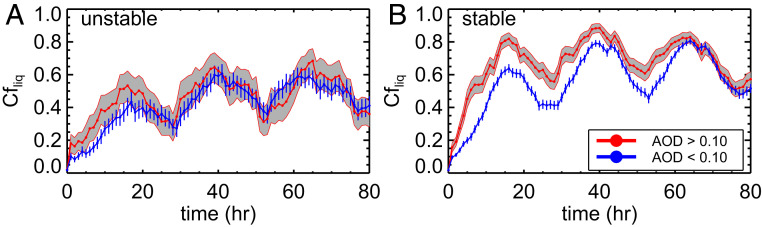
Time series of the CERES SYN cloud fraction for the global-scale ensemble class of oceanic trajectories spanning the Baja Californian, Chilean, and Namibian regions composited into two stability regimes: (*A*) unstable and (*B*) stable as determined by the initial value (16.5 ± 1.5 and 22.5 ± 1.5 K) and slope over 80 h (1 ± 1 K/d) in LTS for the composite of polluted (red) and clean (blue) cases. A minimum of 30 trajectories are required for each meteorological composite. Uncertainties (shaded region) are represented by the 5th to 95th percentile CIs of the distribution.

This analysis has been extended to two additional stratocumulus cloud decks: those off the coasts of South America and southern Africa (e.g., *SI Appendix*, Figs. S3–S8). In general, similar behavior is observed. $N_{d}$ and cloud fraction are significantly higher at larger AOD. However, *CTH* and liquid water path responses are either insignificant and/or reversed in these locations. Lower *CTH*s with larger liquid water path under absorbing aerosol layers off the coast of Africa have been observed (22), simulated (15), and generally hypothesized to be related to semidirect effects (increased stabilization and lowering of the PBL caused by heating of the atmosphere by absorbing aerosol above the boundary layer). When the lower tropospheric stability (LTS) is high, the cloud fraction response to increased AOD is greatest across the three stratocumulus cloud decks on average (Fig. 5). We explore links to stability in greater detail in the following section.

### Global-Scale Analysis.

This section quantifies the role of lower troposphere stability in aerosol–cloud interactions using an ensemble of several hundred thousand trajectories across the global oceans (*SI Appendix*, Fig. S10). Trajectories are not constrained by direction but rather, by LTS. Since LTS primarily drives cloud fraction for marine stratus and cumulus cloud regimes (13), meteorological sorting is based on both its average value over the trajectory and its slope over time. Here, we select two initial starting values (16.5 ± 1.5 and 19.5 ± 1.5 K) for a range of slopes on the rate of change in LTS over the trajectories (−2, −1, 1, 2 ± 1 K/d), thereby providing eight meteorological regimes. For reference, the median initial value and slope of the LTS for the Baja Californian stratus region is 19.7 K and −1.5 K/d, respectively.

Again, robust increases in cloud fraction (*SI Appendix*, Fig. S11) and $N_{d}$ (*SI Appendix*, Fig. S12) manifest in polluted trajectories, however, only under stable meteorological conditions. The dependence of LTS on the cloud fraction response to increased AOD has been observed in other studies (23, 24) with the caveat that some uncertainties involving aerosol humidification and increased cloud detection at high AOD may drive an artificially strong AOD cloud fraction response. The liquid water path response to increased AOD has been shown to depend on the cloud base height (25), precipitation state (nonraining or raining), and meteorology (23, 26). As the atmosphere becomes more unstable, the PBL often becomes deeper (27) due to stronger updrafts. These updrafts produce more cumuliform-type clouds that may be more susceptible to an evaporation–entrainment feedback (25) if they are nonprecipitating, causing them to burn off and lead to decreases in liquid water path and cloud fraction (6) as aerosol concentrations increase. However, if the clouds are precipitating, an increase in aerosol concentration can lead to increases in liquid water path due to the suppression of drizzle (23, 26). As the atmospheric stability and capping inversion strength increase, the importance of collision–coalescence and of the evaporation–entrainment feedback weakens, and aerosol perturbations on decreasing water path become smaller (26). A drier free troposphere can also result in greater entrainment drying. Here, the mean relative humidity values at 850 hPa are about 20% lower in unstable trajectories, which may partially explain the lack of a strong decrease in cloud fraction and liquid water path in the unstable regime. Our results suggest that moistening by aerosol-induced drizzle suppression may be larger than the evaporation–entrainment feedback, thereby allowing the clouds to redistribute cloud water horizontally and increase cloud cover fraction under stable atmospheric conditions.

Total radiative forcing (Eq. **3**) is calculated for each regime separately with mean values displayed in Table 1 (and plotted in *SI Appendix*, Fig. S13). Aerosols significantly increase cloud albedo and fraction particularly under stable atmospheric conditions during the early development of cloud formation along the trajectories. The Twomey effect comprises the bulk of the radiative forcing estimate consecutively for each day along the trajectories (over 50%) due to the large increase in $N_{d}$ (*SI Appendix*, Fig. S12). The average radiative forcing attributed to aerosol–cloud interactions is 0.61±0.34 W/$m^{2}$, which is in quantitative agreement with the estimates from other satellite-based studies (26, 28). Note that the total radiative effect is more than two times larger in the high-stability regimes partly owing to increases in cloud fraction compared with the low-stability regimes. Interestingly, the liquid water path response is weak and can be either negative or positive, which is in general agreement with several recent studies (26, 28–30). These observations support our assumption that strong temperature inversions limit dry air entrainment into the PBL, thereby enhancing the longevity of polluted clouds in stable meteorological regimes.

**Table 1.** Total aerosol radiative forcing decomposed into Twomey, liquid water path, and cloud fraction components using Eq. 3 from thousands of trajectories initialized over global oceanic areas from $60^{○}$S to $60^{○}$N averaged over eight meteorological LTS regimes

Forcing (W/$m^{2}$)	Day 1	Day 2	Day 3
Twomey	−0.34±0.28	−0.42±0.31	−0.61±0.41
Liquid water path	−0.06±0.03	+0.09±0.05	−0.04±0.06
Cloud fraction	−0.25±0.08	−0.08±0.03	−0.08±0.03
Total	−0.66±0.30	−0.42±0.31	−0.76±0.42

### Cloud Lifetime Effect.

These results demonstrate that aerosols have a nonnegligible effect on cloud fraction in the stratus-to-cumulus transition zone. Do aerosols enhance cloud lifetime? We introduce two timescales to seek an answer to this question: 1) cloud-formation timescale as defined by the amount of time it takes to increase cloud fraction over a $1^{○}\times 1^{○}$ region from 0 to over 0.75 ($\tau_{f}$) and 2) cloud-persistence timescale defined as the amount of time the cloud fraction remains above 0.75 for a maximum period of 24 h ($\tau_{p}$). Several threshold values were examined; however, we selected these values as a means to optimize the total number of samples in this study. These conditions are met in approximately 80% of trajectories (i.e., cloud fraction remains below this threshold in the remaining cases). An example of a three-day trajectory off the coast of Africa in which the formation timescale is approximately 15 h (first occurrence where cloud fraction increases above 0.75) with 24-h persistence is displayed in *SI Appendix*, Fig. S14.

**Fig. 6.**
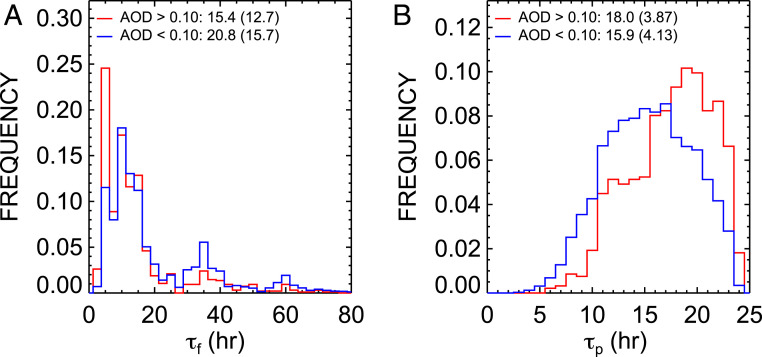
Histogram of timescales for (*A*) cloud formation ($\tau_{f}$) and (*B*) 24-h persistence ($\tau_{p}$) for the class of trajectories as observed by CERES SYN between 2008 and 2017 for the combined trajectories off the coasts of Baja California, Chile, and Africa for clean and polluted trajectories as determined by an AOD threshold of 0.10. Means and SDs (in parentheses) are provided.

From the ensemble of trajectories off the coasts of Baja California, Chile, and Namibia, it takes approximately 20 h on average for cloud fraction to exceed 0.75 along trajectories (Fig. 6*A*). Interestingly, the cloud fraction threshold of 0.75 is reached roughly 5 h earlier when the atmosphere is polluted (AOD>0.10) despite having similar meteorological conditions. An explanation for the shorter cloud-formation timescale may be due to faster destabilization and moistening of the dry boundary layer from enhanced long-wave radiative cooling rates of polluted clouds with larger droplet concentrations (4). However, larger values of AOD may also contain more moisture commonly found in biomass burning plumes (31) that is missed in the European Center for Medium Range Weather Forecasts (ECMWF) Reanalysis (ERA) Interim (ERA-Interim) product or from missing cloud contamination in the satellite AOD product despite the use of state-of-the-art methods (32) to reduce it (*Trajectory Setup*).

Regardless of the formation timescale, clouds also tend to live longer at higher AOD. Fig. 6*B* shows the histogram of the cloud-persistence timescale. Our results are broadly consistent with the Lagrangian decorrelation timescales described in Eastman et al. (33). On average, the cloud-persistence timescale is 2 h longer under polluted conditions. The differences were found to be significant at the 95th percentile using a two-tailed *t* test. We conclude that the larger cloud fraction associated with the polluted trajectories may manifest from the longer cloud lifetimes compared with unpolluted clouds.

This analysis has been extended to all regions and meteorological composites from the trajectories spanning the global oceans (*SI Appendix*, Fig. S15). Slight differences in the timescales are observed between stratocumulus basins (e.g., polluted clouds live longer by about 1 h in Namibia compared with Baja California). The differences may be attributed to stronger shortwave heating by absorbing aerosols and slightly larger LTS and amounts of planetary boundary-layer moisture for the polluted conditions in the Namibia region. In general, aerosols increase cloud lifetime, with the largest increases under stable atmospheric conditions in the main stratus-to-cumulus transition zones.

## Conclusions

This study quantifies aerosol radiative forcing using a combination of geostationary satellite observations and Lagrangian trajectories. Polluted clouds are found to have larger cloud fraction, lifetime, and droplet concentrations under stable atmospheric conditions. These responses are robust across three separate oceanic basins and in several thousand trajectories stratified by gradients in lower troposphere stability, a meteorological variable that is a controlling factor on cloud fraction (11, 13). Evidently, clouds forming in high AOD conditions have markedly larger $N_{d}$ under stable atmospheric conditions. These perturbations persist multiple days and lead to a significant radiative forcing-based estimate that has recently been suggested for warm boundary-layer clouds (34). However, the radiative forcing estimate in unstable atmospheric conditions is markedly smaller by comparison, presumably due to positive feedbacks between dry free-tropospheric air and entrainment (4, 6).

Large eddy simulation experiments following Lagrangian trajectories could be a useful tool to examine why cloud fraction exceeds a threshold of 0.75 earlier under high AOD conditions along Lagrangian trajectories. We propose two hypotheses based on physical mechanisms: 1) the enhanced radiative cooling rates from polluted shallow clouds destabilize and moisten the PBL more rapidly than unpolluted clouds if the air above the boundary layer is sufficiently moist (4); 2) biomass burning plumes may increase boundary-layer humidity in addition to AOD due to their moisture content (31), and this increase in humidity may be missing from the ECMWF ERA-Interim data or from the evaluation of cloud contamination despite efforts to prevent this (32) (*Trajectory Setup*). Another possibility is the increase in subpixel cloud albedo may trigger earlier overcast cloudy pixel detection in the MODIS retrieval algorithm (19) than optically thinner clouds forming in unpolluted conditions.

Solar-absorbing aerosol layers above the boundary layer may result in semidirect effects along some of the trajectories in this study. Observations from NASA’s Cloud Aerosol Transport System 1,064-nm lidar indicate that about 60% of the above-cloud aerosols off the coast of Africa are found within 360 m to the top of the underlying cloud (35). The sensitivity of low cloud cover change to elevated aerosols layers increases as the cloud–aerosol gap decreases (31). Furthermore, aerosol layers observed above the cloud top have less of an influence on decreasing cloud droplet size compared with when the aerosol–cloud layers touch (36). However, despite these potential semidirect effects off the coast of Africa, the general cloud responses to increased AOD are remarkably consistent in all three regions examined in this study.

The stratus-to-cumulus transition is not, to first order, driven by changes in precipitation and aerosol concentration but by slowly varying gradients in sea surface temperature and large-scale subsidence (13). On average we find that aerosols increase cloud fraction along Lagrangian trajectories in stable atmospheric conditions. The extent to whether aerosols influence cloud morphology along the classic stratus-to-cumulus transition zone remains an open question. This transition zone contains a rich variety of cloud regimes (for example, open and closed cellular clouds displayed in Fig. 1) that manifest through rapidly changing processes like precipitation. Thermodynamic variables such as mixing ratio, cloud base height, and precipitation adjust on fast timescales (typically about 12 h) compared with the timescale of the inversion height that is set primarily by large-scale divergence (2 to 5 d) (37), which can take place gradually over the stratus-to-cumulus transition zone. It has been hypothesized that suppressing precipitation can transform broken open cellular cloud decks along this transition zone into closed overcast clouds. Such cloud regime transitions lead to significant local cloud radiative effects (38) and hence, may be critical for the development and maintenance of the stratus-to-cumulus transition. Future work linking Lagrangian trajectories to higher-resolution satellite imagery and cloud classification [e.g., based on machine learning techniques (39)] may provide deeper clues to the role of aerosol on the stratus-to-cumulus transition and climate change.

## Materials and Methods

### Data.

The CERES SYN Ed 4 product provides global temporally and spatially resolved aerosol, cloud, and radiation products. This product condenses petabytes of geostationary satellite data into a user-friendly gridded format ($1^{○}$ latitude by $1^{○}$ longitude resolved hourly) over the 2003 to 2018 period. The product is well suited to study the diurnal cycle of cloud properties and radiative fluxes in diverse locations over land and ocean (16, 40). This product uses 16 geostationary satellites. To ensure consistency across the various satellite sensors, cross-calibration of the instrument channels is tuned to match the cloud optical property retrievals from the MODIS collection 5 product (41). Top and bottom of atmosphere broadband radiative fluxes are calculated using the Langley Fu-Liou radiative transfer model using MODIS-assimilated aerosol retrievals in the Model for Atmospheric Transport and Chemistry and meteorological data from the Goddard Earth Observing System. Broadband radiative fluxes are normalized against the single-scanning footprint observations from CERES data acquired from Terra and Aqua satellites. The Langley Fu-Liou Ed. 4 model provides temporally resolved fluxes, which are used in the CERES Energy Balanced and Filled products (42). The monthly mean uncertainties are based on 37 land and 48 ocean buoy locations; the surface net shortwave and long-wave fluxes are 5.7 and 2.9%, respectively (16).

High-spatial resolution geostationary imagery data (3 km at the subsatellite point with a repeat cycle of 15 min) from SEVIRI on the Meteosat Second Generation satellite with 12 spectral channels (0.64, 0.84, 1.6, 3.9, 6.2, 7.3, 8.7, 9.7, 10.8, 12.0, 13, and high resolution visible broadband 0.4–1.1 μm) are used to retrieve cloud properties from the NASA Langley cloud retrieval for the ObseRvations of Aerosols above Clouds and their intEractionS (ORACLES) campaign. This algorithm uses the same architecture of the CERES SYN products. Due to limitations in data volume, we could only process the period during the ORACLES campaign to study the evolution of a POC (described by Fig. 1).

MODIS onboard Terra and Aqua satellites contain 36 spectral channels. The Terra satellite is in a descending node of the orbit crossing the equator at 10:30 and 22:30 local time. Similarly, Aqua is in an ascending node crossing the equator at 13:30 and 01:30 local time. Cloud and aerosol optical properties (cloud optical thickness, cloud effective radius) are provided twice per day in the sunlit part of the orbit, while *CTH*, pressure, and fraction are provided at nighttime too in the collection 6.1 products.

Precipitation is obtained from the IMERG V06B product, which combines passive microwave measurements from the Global Precipitation Measurement mission, observations from polar orbiting Advanced Microwave Scanning Radiometers, brightness temperature measurements from multiple satellites in geostationary orbit, and rain gauge data. The gridded product (0.$25^{○}$) provides precipitation retrievals every half hour on a 0.$1^{○}$ grid from 2015 to 2018, making it ideal to quantify precipitation changes along trajectories.

The narrow swath (effective footprint of 1.4×1.8 km) and limited temporal sampling of the w-band CloudSat cloud-profiling radar do not permit a detailed examination of precipitation in a Lagrangian framework. However, we use it here to quantify precipitation responses at 1:30 PM (only daytime observations are available in this study due to a battery failure in 2011) from the 2C-Column-Precip product (43). While CloudSat is more sensitive to detecting light precipitation (minimum detectable signal is −28 dBZ) compared with passive radars that make up the IMERG product (minimum detectable signal is approximately +15 dBZ), *SI Appendix*, Fig. S16 shows supporting evidence that less frequent occurrence of precipitation is identified under polluted conditions (AOD>0.1) in both datasets.

Meteorological variables from the ECMWF ERA-Interim products are collocated to each trajectory; these include sea surface temperature, planetary boundary-layer specific humidity, and lower troposphere static stability ($LTS=\Theta_{P700}-\Theta_{Ps}$, where $\Theta_{P700}$ and $\Theta_{Ps}$ are the potential temperatures at 700 hPa and surface pressure levels, respectively).

### Trajectory Model.

Trajectories are calculated using HYSPLIT (44). The trajectory model uses Air Resources Laboratory-generated Global Data Assimilation System (GDAS) meteorological data. GDAS outputs analysis time steps six times daily at 0.$5^{○}$ horizontal spatial resolution with 55 hybrid sigma-pressure levels. Each forward trajectory begins at 13:30 local time to coincide with the multisensor observations from the A-Train. Forward trajectories are run for a duration of 80 h to represent the average amount of time required to transition from stratus to cumulus (13).

To ensure that trajectories follow the mean motions of the PBL, they are initialized in the middle of the PBL (determined by the thermodynamic sounding) and constrained to flow along an isobaric surface to avoid escaping into the free troposphere. The HYSPLIT model as shown in Fig. 1 (Movie S1) shows its seamless ability to track boundary-layer clouds over multiple days. The depth of the PBL is determined within HYSPLIT using the profiles of temperature, humidity, and wind velocity (44). Uncertainties associated with the particle trajectory positions are typically larger near converging winds and/or frontal systems. The uncertainty scales approximately 20% with distance from origin. Hence, for an 80-h period covering roughly 1,500 km along relatively stable subtropical atmospheric conditions, we estimate that the maximum displacements due to particle trajectory uncertainty are about 300 km in this study.

### Trajectory Setup.

All forward trajectories are initialized over the ocean in clear-sky conditions as determined by the high-confidence cloud mask flag from the Aqua MODIS collection 6.1 (MYD08) product. Initializing in clear-sky conditions provides 1) an accurate baseline *AOD*, 2) known history of the aerosol-containing air mass prior to cloud formation, 3) a baseline cloud radiative effect for quantifying cloud system evolution, 4) a starting point to track the initial development and timescale of cloud formation and dissipation, and 5) ability to sort between clean and polluted trajectories. The clear-sky retrievals are defined by cloud fraction (over a $1^{○}\times 1^{○}$ degree region) being less than 5% and intercloud spacing being greater than at least 15 km. This criterion is established with the aim to reduce retrieval errors in high-cloud fraction scenes related to aerosol humidification from swelling near cloud edges, aerosol brightening due to 3D scattering from the sides of nearby clouds, and retrieval artifacts due to the presence of cloud contamination (32). The AOD typically decreases away from cloud until leveling off at a length scale of approximately 15 km. The usage of potentially contaminated AOD retrievals located within 15 km from nearby cloud significantly enhances the aerosol indirect effect (32). However, the gridded daily level 3 MODIS *AOD* product can be corrected following the above criteria to yield the statistically similar radiative forcing-based estimates compared with using high-resolution level 2 cloud and aerosol paired pixels. Therefore, our analysis restricts the use of gridded AOD estimates in regions largely devoid of clouds and over dark ocean surfaces to increase the accuracy of the AOD through limiting the possibility of cloud contamination and humidification. It is noteworthy that sometimes a significant portion of the AOD can reside above the boundary layer, particularly for the Namibia region, and thereby contribute to greater uncertainty in the classification of clean and polluted conditions. Overall, these initial conditions provide the unique ability to link the history of the aerosol to the developing cloud system. Only the warm-cloud process is considered here; therefore, forward trajectories are rejected if they contain any multilayer cloud layers, ice cloud, or high-level cloud (cloud top pressure less than 500 hPa).

Hydroscopic growth results in greater attenuation of the incoming solar radiation than for the same number concentration of dry aerosol particles. To ensure that the clean and polluted trajectories are not influenced by the planetary boundary-layer humidity, we have examined the AOD based on dry conditions (relative humidity =30%) using the Copernicus Atmosphere Monitoring Service (CAMS) reanalysis product. Mixing ratios of each aerosol constituent (CAMS aerosol composition variables: sea salt, dust, organic matter, black carbon, and sulfate) is provided for 25 vertical levels. Column integrated dry-mode *AOD* is calculated following $\tau =\sum_{n=1}^{N}\int_{p_{s}}^{0}\beta_{e}r\left(p\right)dp/g$, where N is the total number of aerosol species, $\beta_{e}$ (meters^2^ per gram) is the dry-mass extinction coefficient for each aerosol type using the look tables described in Benedetti et al. (45), r(p) (grams per kilogram) is the mass mixing ratio of the aerosol species at each vertical level, g (meters per second^2^) is the constant of gravity, dp is the pressure of the model level, and $p_{s}$ is the surface pressure. *SI Appendix*, Fig. S17 shows a comparison between CAMS AOD and CAMS dry-mass AOD for the composite of trajectories initialized off the coast of California. On average, dry-mass AOD is approximately 30% smaller than the assimilated AOD due to smaller mass extinction coefficients and less hydroscopic growth when the air is dry. However, the strong correlation coefficient ($r^{2}=0.91$) between dry and wet *AOD* implies that humidification effects are essentially linear and roughly the same between clean and polluted conditions as determined by MODIS satellite observations. We have taken this analysis a step further by using dry *AOD* as the proxy for our aerosol-cloud interaction metrics (instead of MODIS) and find similar results.

#### Radiative Forcing.

Aerosol radiative forcing is calculated based on the method following Quaas et al. (46) in which the forcing is decomposed into a Twomey effect and adjustments to Twomey are based on changes in liquid water path and cloud fraction associated with an increase in *AOD*. The CERES SYN product is used to calculate terms in the following equation:

$$\frac{\Delta \alpha }{\Delta \ln AOD}=C\Delta \alpha_{c}\left(1-\alpha_{c}\right)\frac{\Delta \ln N_{d}}{\Delta \ln AOD}\left(\frac{1}{3}+\frac{5}{6}\frac{\Delta \ln L}{\Delta \ln N_{d}}+\frac{\Delta \ln C}{\Delta \ln N_{d}}\right),$$ [1]

where C is the cloud fraction over the spatial domain, $\alpha_{c}$ is the average cloud albedo, $N_{d}$ is the cloud droplet concentration, L is the liquid cloud water path, and AOD is the aerosol optical thickness. Liquid water path is calculated using $L=2/3\rho_{l}r_{e}\tau_{c}$ (47), where $r_{e}$ is the satellite-retrieved droplet effective radius and $\tau_{c}$ is the cloud optical thickness. The Twomey, liquid water path, and cloud fraction radiative effect contributions are shown as the first three terms in parentheses of Eq. **1**. The $N_{d}$ is calculated as

$$N_{d}=\gamma \sqrt{\tau_{c}}r_{e}^{-2.5},$$ [2]

where γ=1.37e−5 $m^{-0.5}$ is related to the adiabatic condensation growth rate and adiabaticity factor typical for stratocumulus clouds. Grosvenor et al. (47) can be consulted for a comprehensive assessment of the uncertainties. Cloud property differences are calculated from the population of polluted and clean trajectories. Composites are based on the AOD at the start of each trajectory within the clear-sky footprint of the satellite sensor. A value of 0.10 was chosen as it is the median value of the global AOD distribution (*SI Appendix*, Fig. S2). Our estimate of aerosol radiative forcing is calculated by multiplying Eq. **1** with the incoming solar radiation and anthropogenic *AOD* following the method described in ref. 48 as

$$\Delta F_{TOA}=-F^{↓}\frac{\Delta \alpha }{\Delta \ln AOD}\ln AOD_{anth},$$ [3]

where $F^{↓}$ is the incoming solar radiative flux at the TOA is 340.2 W/$m^{2}$ (49) and $AOD_{anth}$ is the *AOD* attributed to anthropogenic activities. $AOD_{anth}$ is obtained from the ECMWF Monitoring Atmospheric Composition and Climate (MACC) II product. Natural anthropogenic aerosol contributions are provided by the integrated forecast system model constrained by satellite-retrieved AOD at a wavelength of 0.55 μm from MODIS. On average, this leads to an anthropogenic aerosol fraction of approximately 20% globally (for example, see ref. 48) (Fig. 2). Since MACC-II was only produced from 2003 to 2012, we use an annual climatology for this period to represent the spatially averaged anthropogenic aerosol concentration.

### Data and Code Availability.

CERES SYN Ed4a 4 product is available at https://ceres.larc.nasa.gov. MODIS collection 6 MYD08_D3 product is available at https://earthdata.nasa.gov. IMERG data are available from the NASA Goddard Space Flight Center (https://pmm.nasa.gov). CloudSat data are available from the Cooperative Institute for Research in the Atmosphere (http://www.cloudsat.cira.colostate.edu). ECMWF data were obtained from https://www.ecmwf.int. HYSPLIT trajectory code is available at https://www.ready.noaa.gov/HYSPLIT.php. All data and code availability websites were last accessed on 3 October 2020.

## Supplementary Material

Supplementary File

Supplementary File
