# Supplementary material for: Aerosols enhance cloud lifetime and brightness along the stratus-to-cumulus transition
Source: Proc Natl Acad Sci U S A. 2020 Jul 13;117(30):17591–8. doi: 10.1073/pnas.1921231117 (PMC7395436; doi:10.1073/pnas.1921231117)
Supplement: Supplementary File [file pnas.1921231117.sapp.pdf]

PNAS
www.pnas.org

Supplementary Information for

Aerosols Enhance Cloud Lifetime and Brightness along the Stratus-to-Cumulus Transition

Matthew W. Christensen, William K. Jones and Philip Stier

Matthew Christensen
E-mail: matthew.christensen@physics.ox.ac.uk

This PDF file includes:

Figs. S1 to S17
Legend for Movie S1

Other supplementary materials for this manuscript include the following:

Movie S1

www.pnas.org/cgi/doi/10.1073/pnas.1921231117

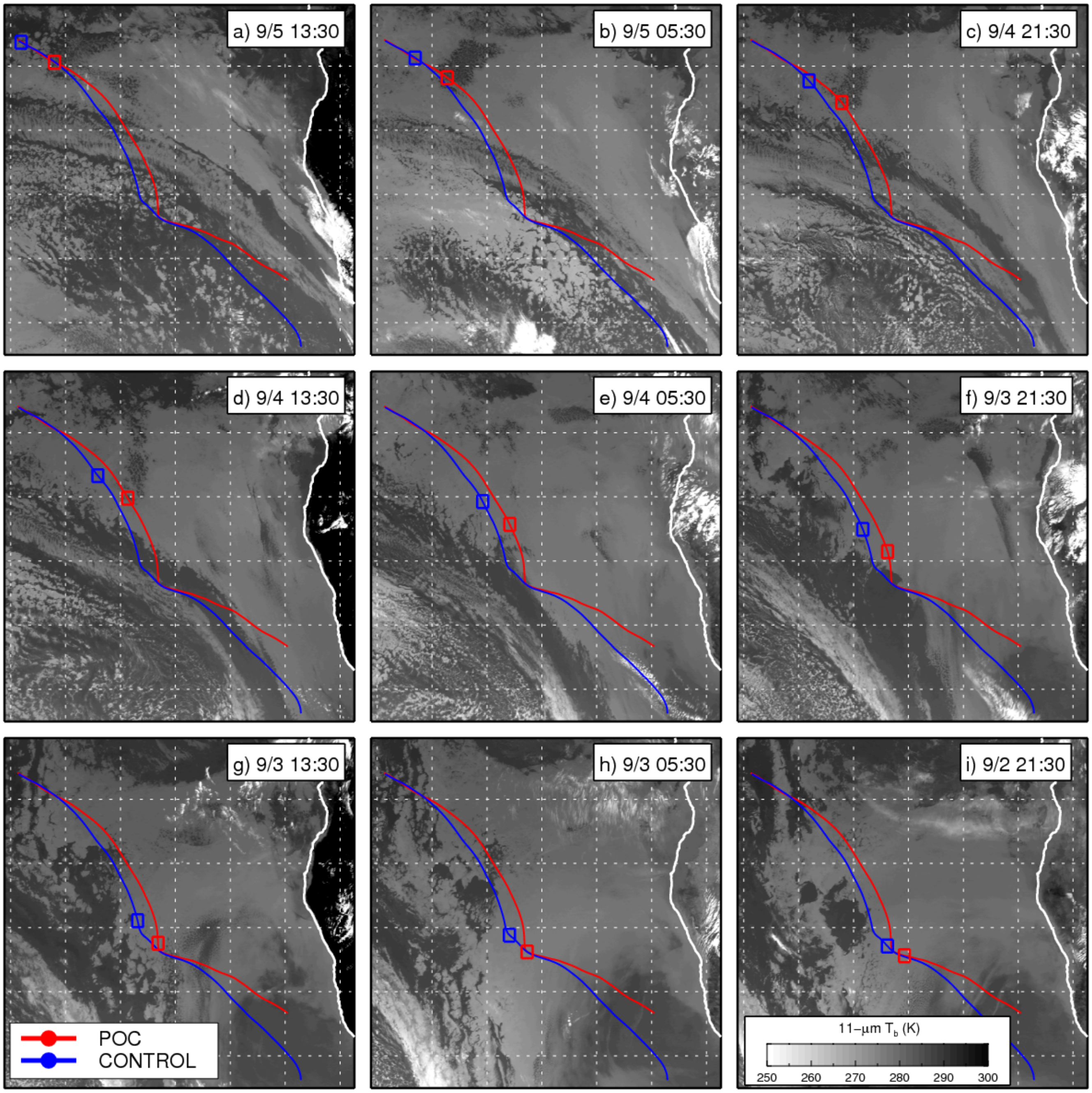

Fig. S1. SEVIRI 11-μm brightness temperature imagery plotted at selected times for the same region and trajectories displayed in Fig. 1a.

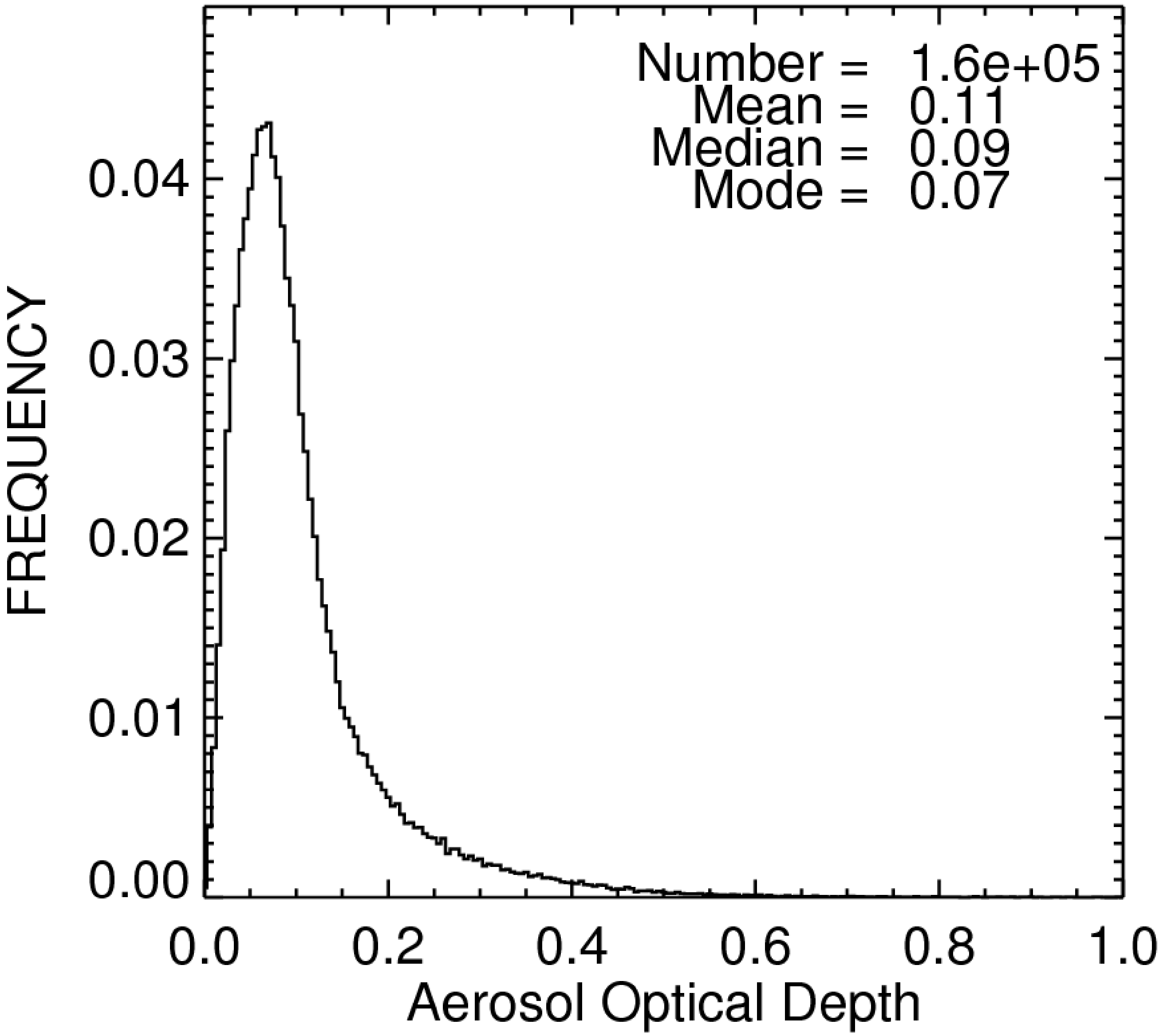

Fig. S2. Histogram of the aerosol optical depth retrieved from CERES SYN at the start of each forward trajectory initialized in clear-sky conditions over oceanic regions from 60° S – 60° N.

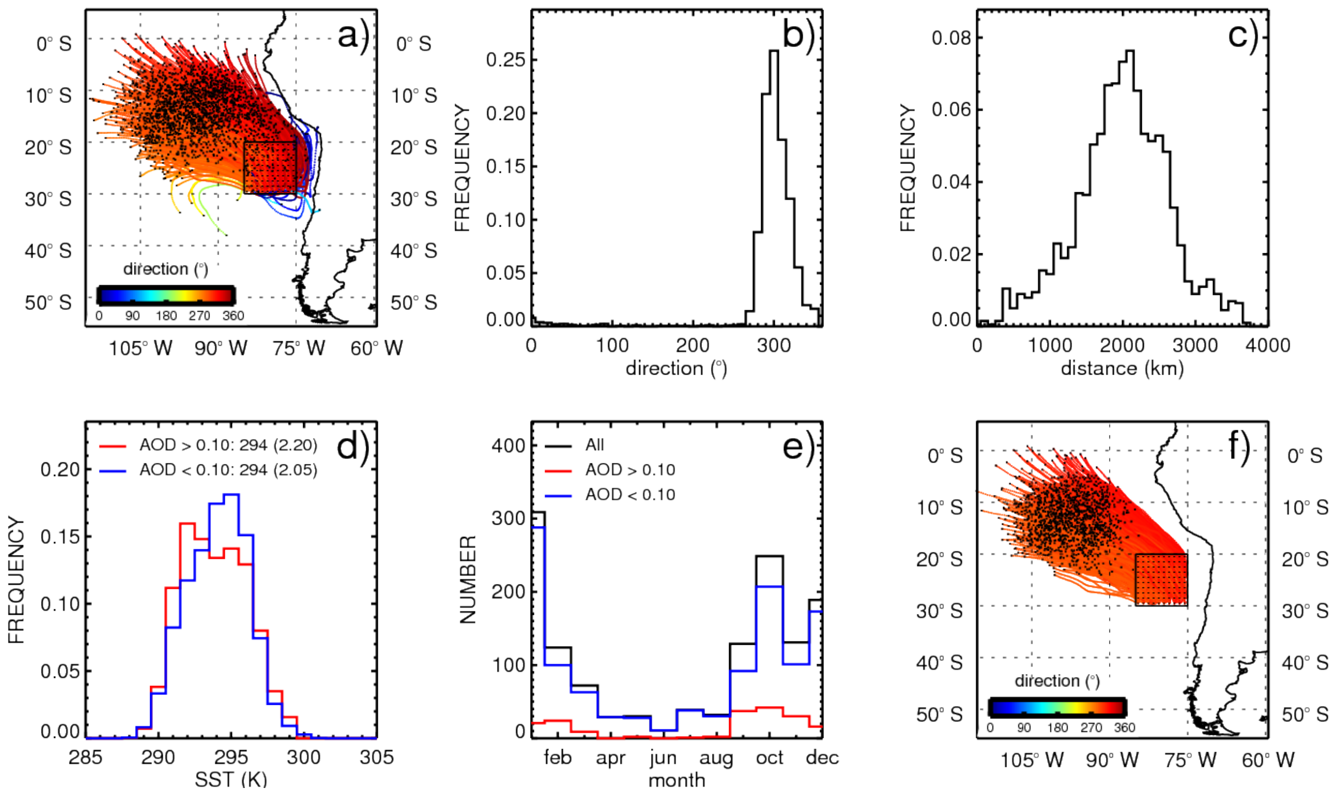

Fig. S3. Ensemble of 80-hr period forward trajectories initialized in clear-sky conditions during the period from 2015 – 2017 off the coast of Chile (85° W – 75° W; 30° S – 20° N). Trajectory direction (southerly direction is defined as 180°) determined from its starting location (boxed region) to the final position is plotted using a rainbow color bar. Histograms of the direction (b) and length (c) are provided for the composite shown in panel a). Trajectories that are constrained to flow along the median wind direction (210 ± 10 degrees) and cover at least 1000 km are displayed in panel f). Histograms of sea surface temperature (SST) and monthly occurrence for unpolluted ($AOD < 0.10$; red) and polluted ($AOD > 0.10$) conditions are shown, respectively (d and e).

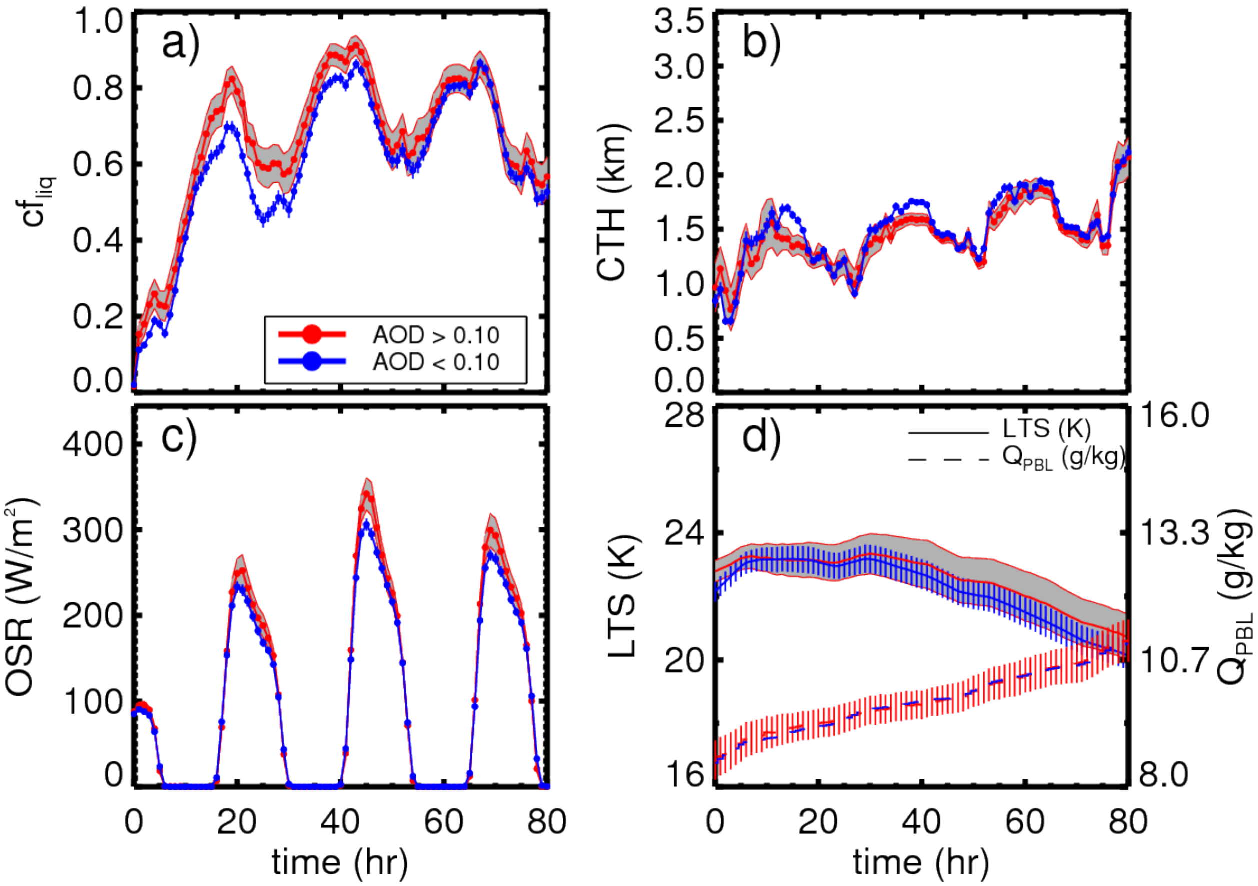

Fig. S4. Time-series of the mean (a) liquid cloud fraction, (b) cloud top height, (c) top of atmosphere (TOA) outgoing shortwave radiative flux from CERES SYN and (d) lower troposphere stability and planetary boundary layer humidity for the composite of polluted (red line) and unpolluted (blue line) trajectories off the coast of Chile. Natural variability (shaded region and vertical bars for the unpolluted clouds) is represented by the 5 – 95th percentile confidence interval of the distribution.

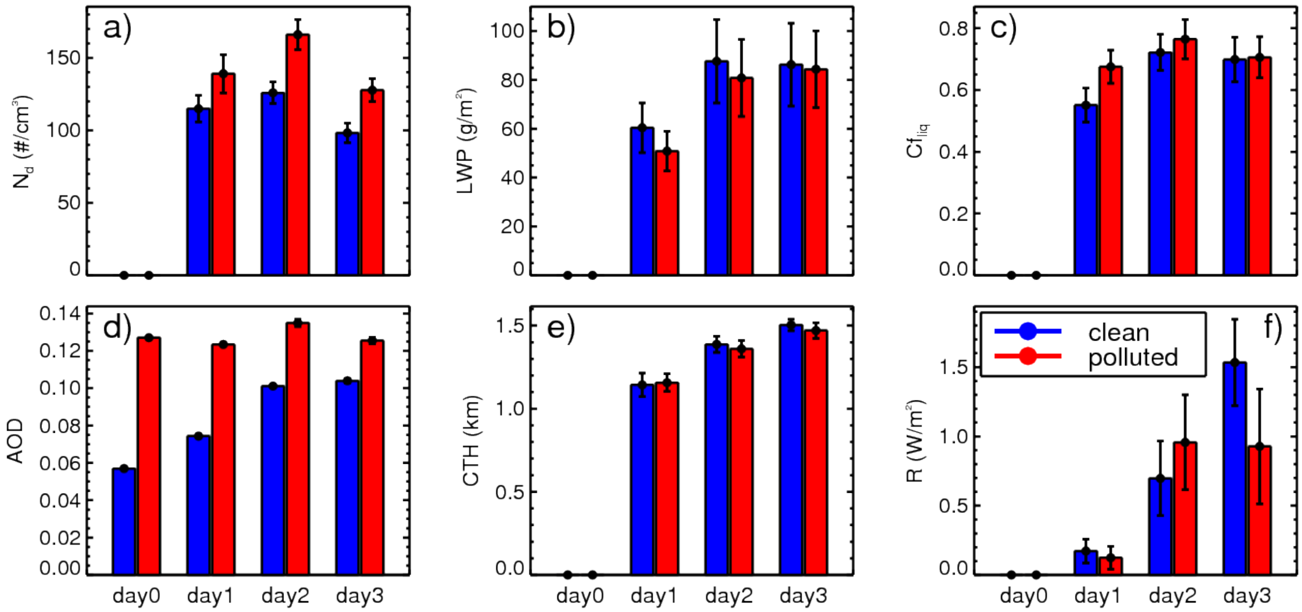

Fig. S5. Day-time average (9am to 4pm local-time) cloud droplet concentration (a), liquid water path (b), cloud fraction (c), CAMS aerosol optical thickness (1:30 pm only) (d), cloud top height (e) and IMERG precipitation rate multiplied by the latent heat of vaporization (i.e. LP) (f) along the ensemble of trajectories off the coast of Chile. The 5 – 95th percentile confidence interval are given by the vertical bars.

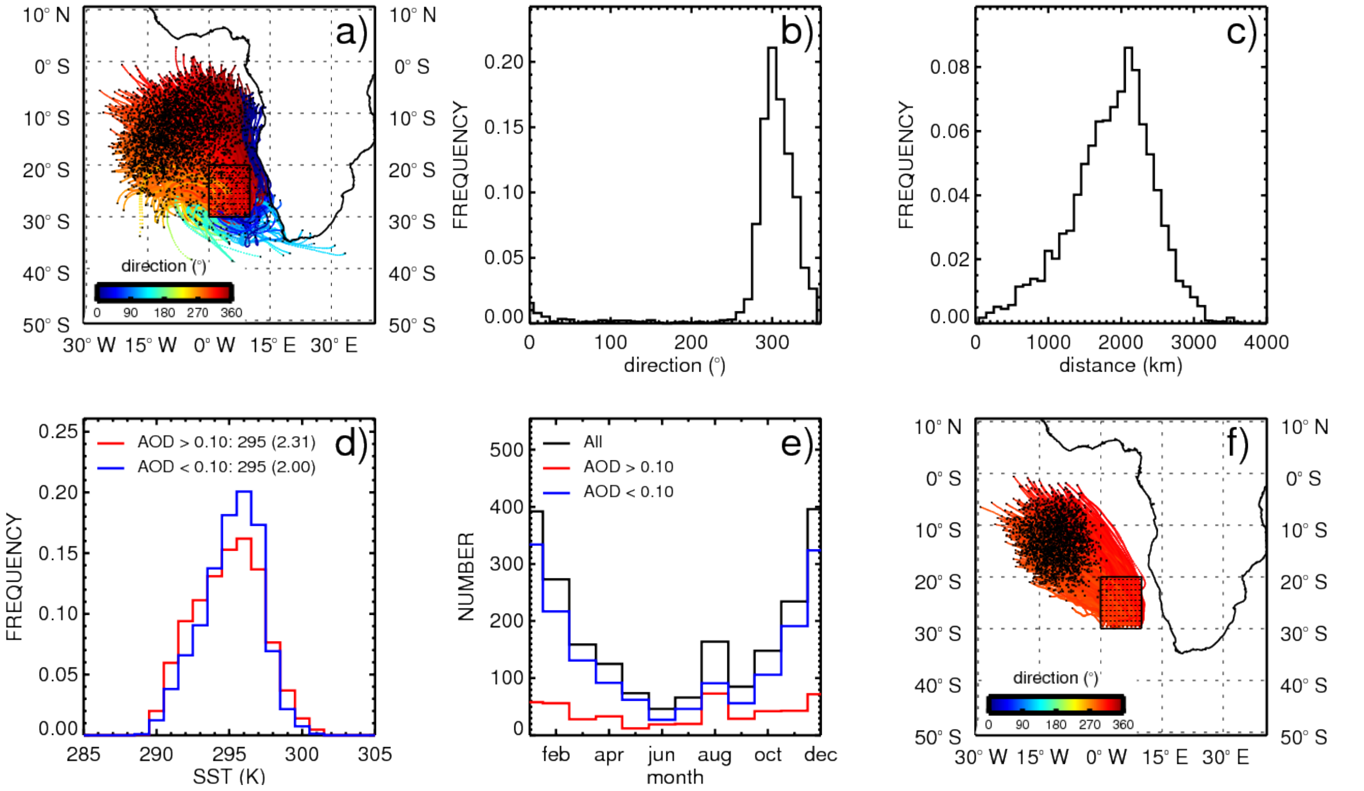

Fig. S6. Ensemble of 80-hr period forward trajectories initialized in clear-sky conditions during the period from 2015 – 2017 off the coast of Namibia (0° E – 10° E; 30° S – 20° N). Trajectory direction (southerly direction is defined as 180°) determined from its starting location (boxed region) to the final position is plotted using a rainbow color bar. Histograms of the direction (b) and length (c) are provided for the composite shown in panel a). Trajectories that are constrained to flow along the median wind direction (210 +/- 10 degrees) and cover at least 1000 km are displayed in panel f). Histograms of sea surface temperature (SST) and monthly occurrence for unpolluted ($AOD < 0.10$; red) and polluted ($AOD > 0.10$) conditions are shown, respectively (d and e).

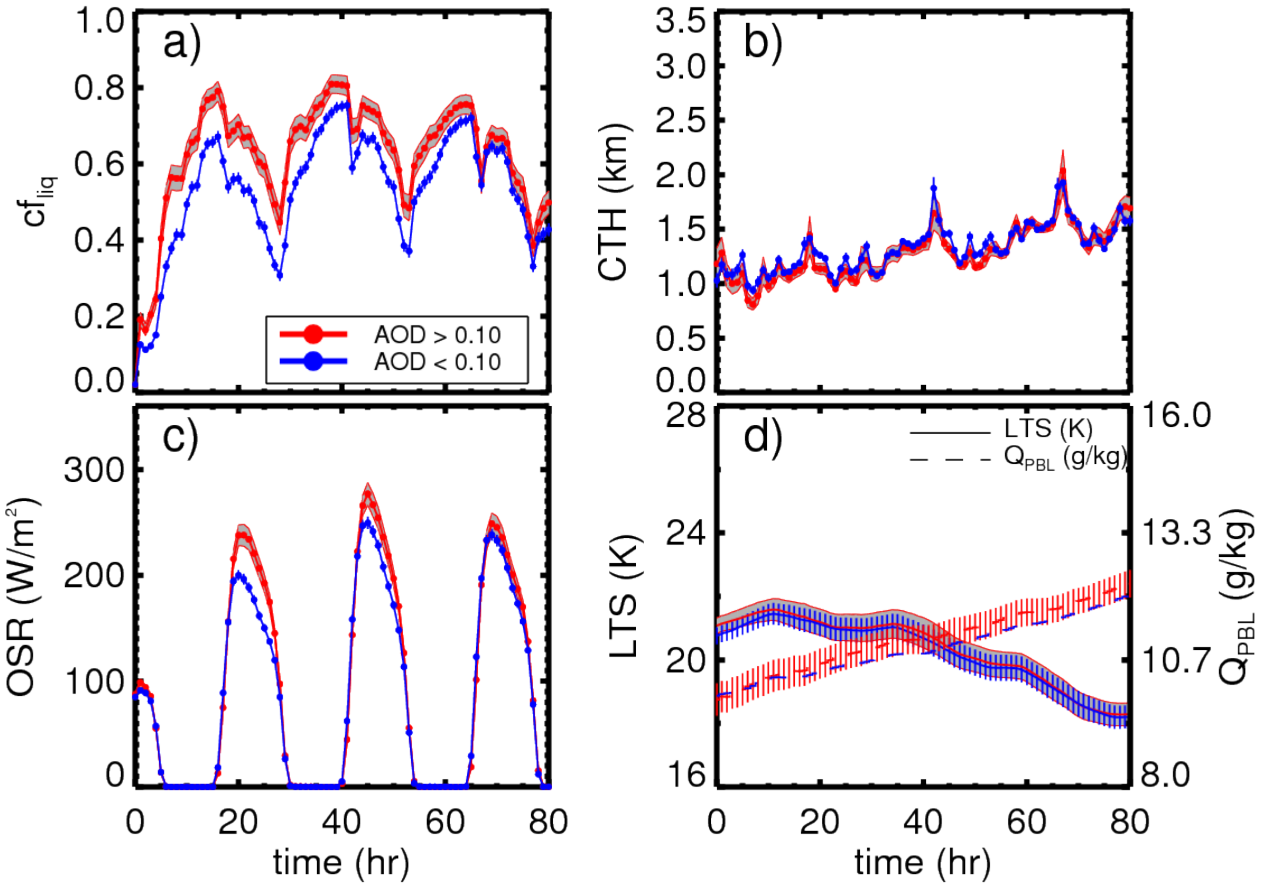

Fig. S7. Time-series of the mean (a) liquid cloud fraction, (b) cloud top height, (c) top of atmosphere (TOA) outgoing shortwave radiative flux from CERES SYN and (d) lower troposphere stability and planetary boundary layer humidity for the composite of polluted (red line) and unpolluted (blue line) trajectories off the coast of Namibia. Natural variability (shaded region and vertical bars for the unpolluted clouds) is represented by the 5 – 95th percentile confidence interval of the distribution.

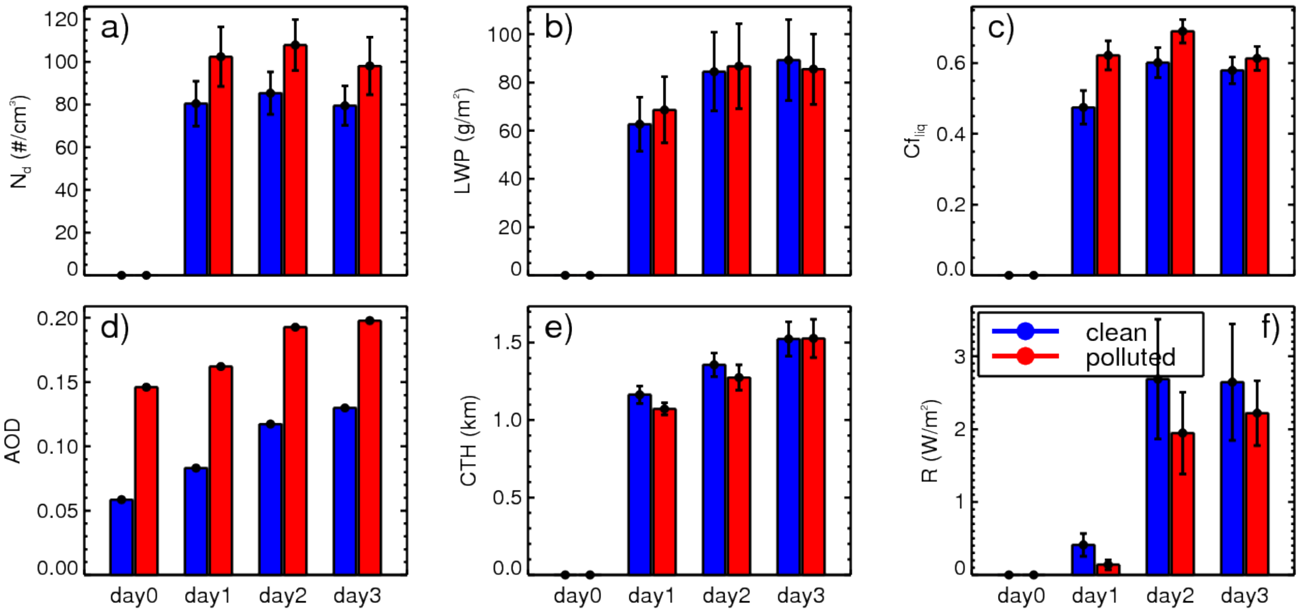

Fig. S8. Day-time average (9am to 4pm local-time) cloud droplet concentration (a), liquid water path (b), cloud fraction (c), CAMS aerosol optical thickness (1:30 pm only) (d), cloud top height (e) and IMERG precipitation rate multiplied by the latent heat of vaporization (i.e. LP) (f) along the ensemble of trajectories off the coast of Namibia. The 5 – 95th percentile confidence interval are given by the vertical bars.

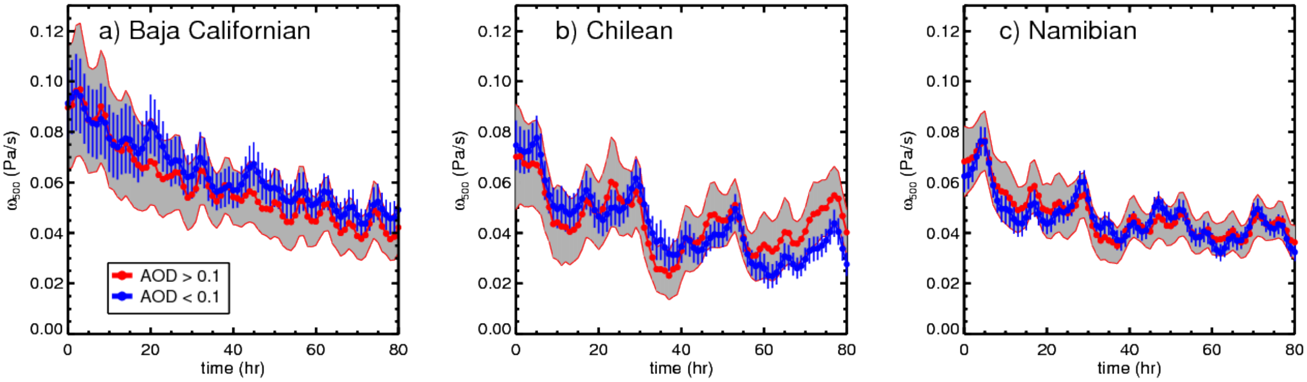

Fig. S9. Time-series of the mean subsidence rate at the 500 hPa pressure level (ω_{500}) for the composite of polluted (red line) and unpolluted (blue line) trajectories off the coasts of a) Baja California, b) Chile and c) Namibia.

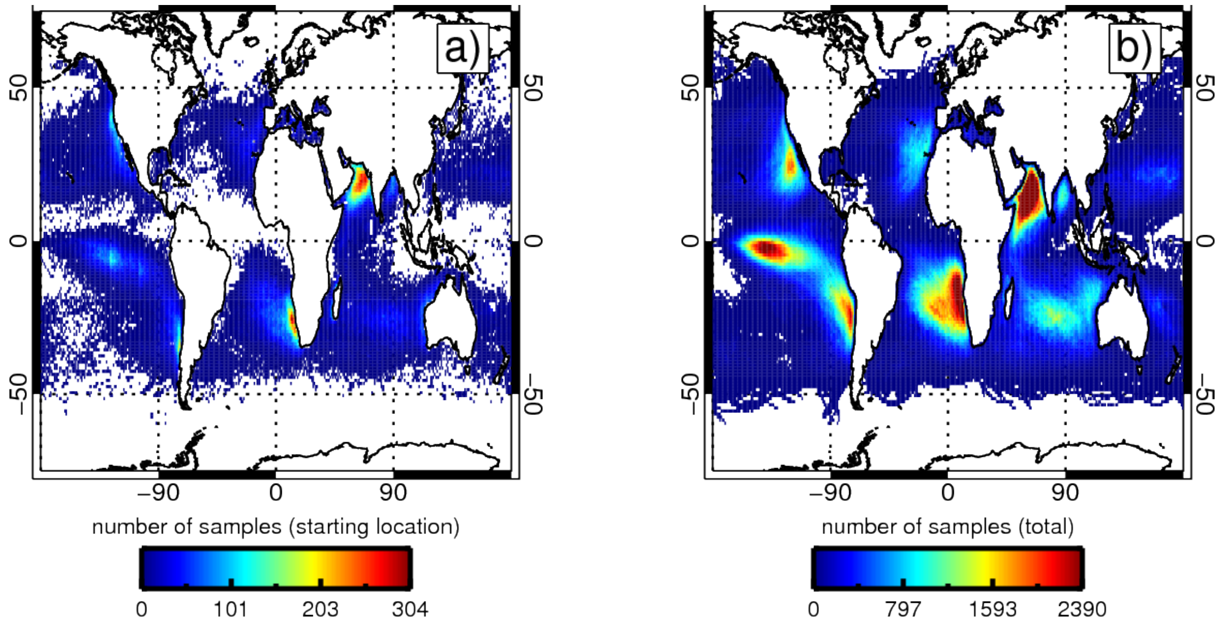

Fig. S10. Distribution of a) initial starting points in clear-sky boundary layers as determined by CERES SYN1deg1hr and b) locations along all trajectories between 60° S – 60° N across oceanic locations.

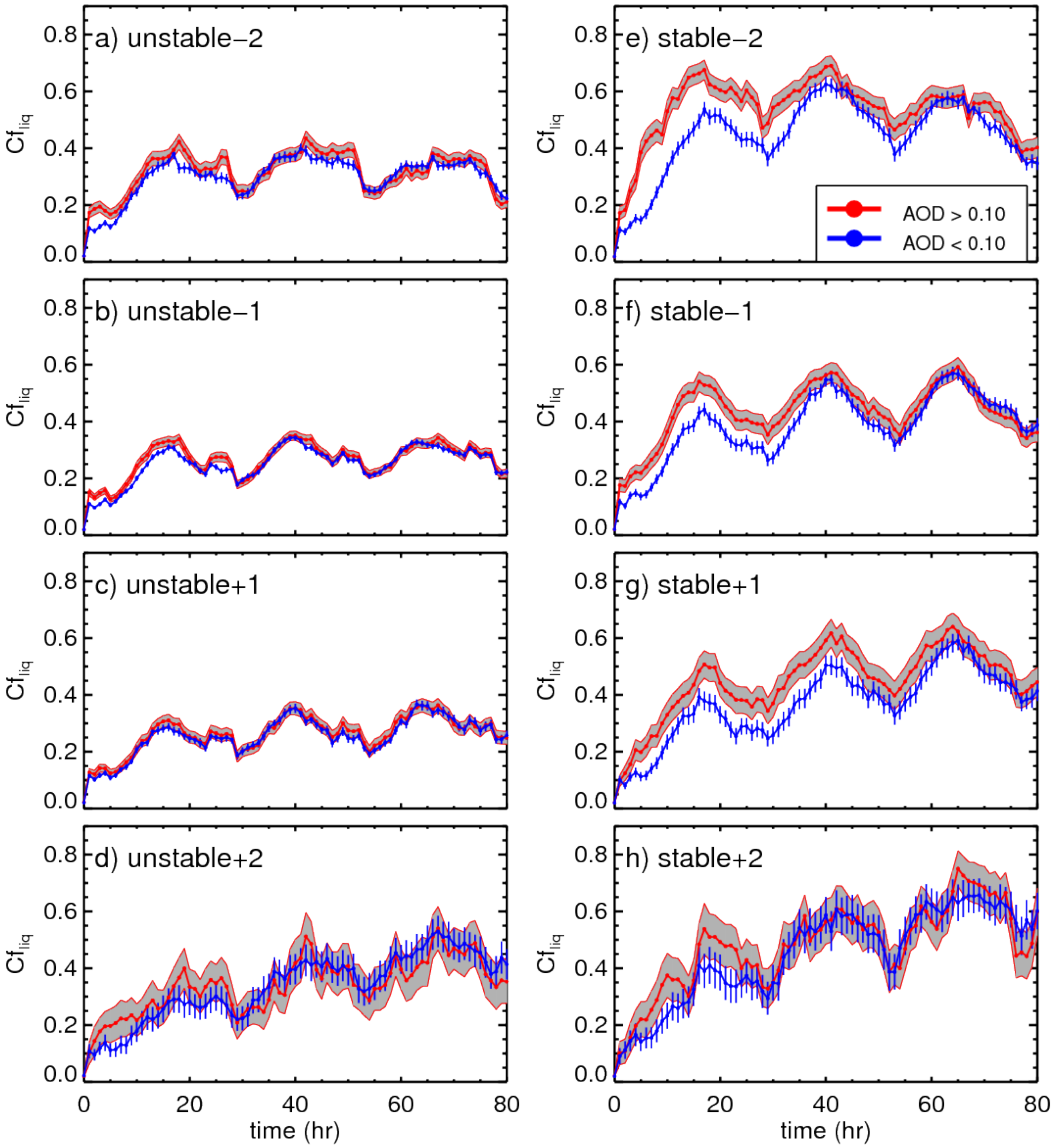

Fig. S11. Time series of the CERES SYN cloud fraction for the global-scale ensemble class of oceanic trajectories spanning 60° S – 60° N composited into the 8 stability regimes determined by the average and slope of the LTS for the composite of polluted (red) and clean (blue) cases. Uncertainties (shaded region) are represented by the 5 – 95th percentile confidence interval of the distribution.

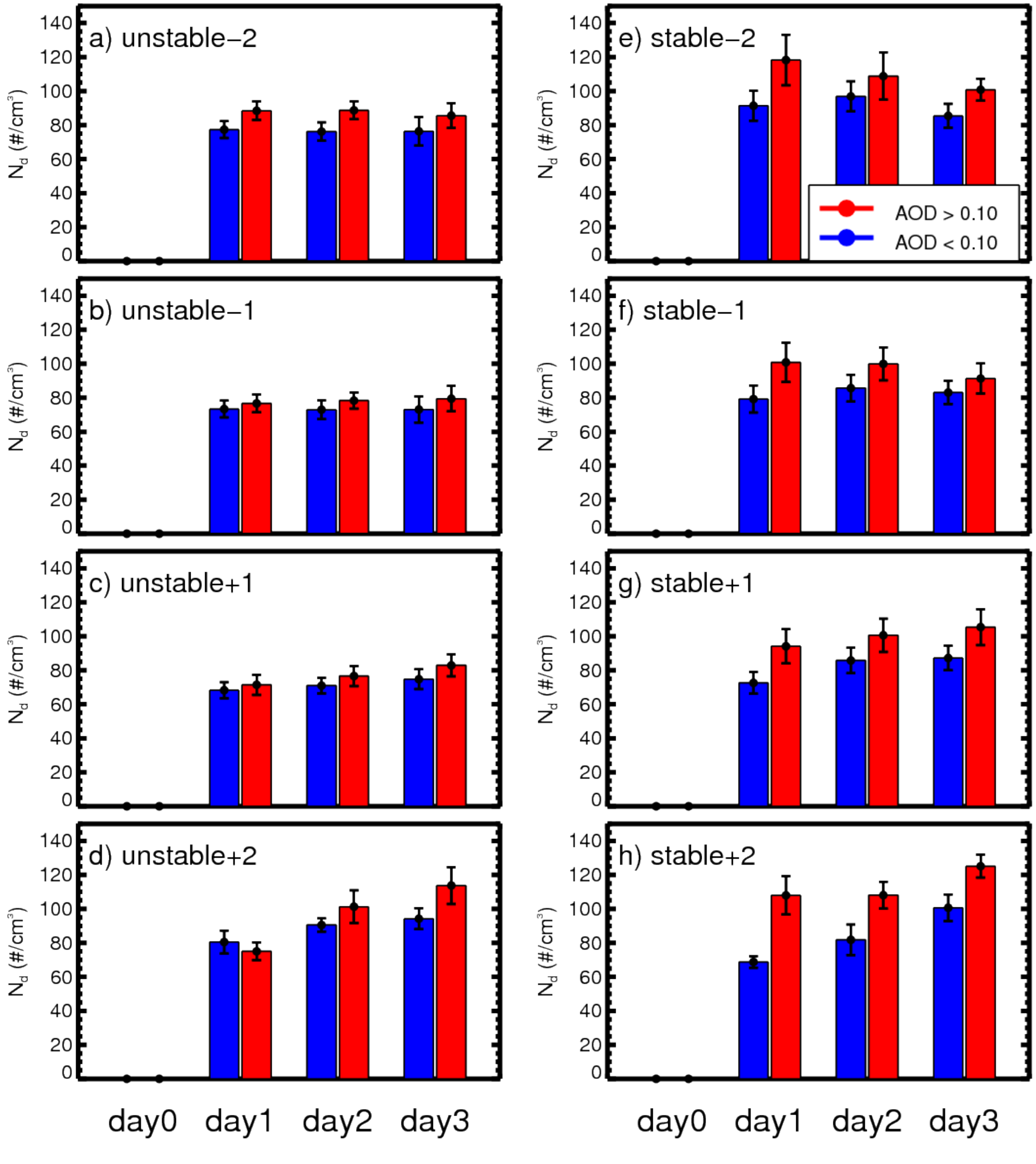

Fig. S12. Day-time average (9am to 4pm local-time) cloud droplet concentration as retrieved from the CERES SYN product for the global-scale ensemble class of oceanic trajectories spanning 60° S – 60° N composited into the 8 stability regimes determined by the mean (LTS) and slope (LTS_m) of the LTS over the trajectory for the composite of polluted (red) and clean (blue) cases.

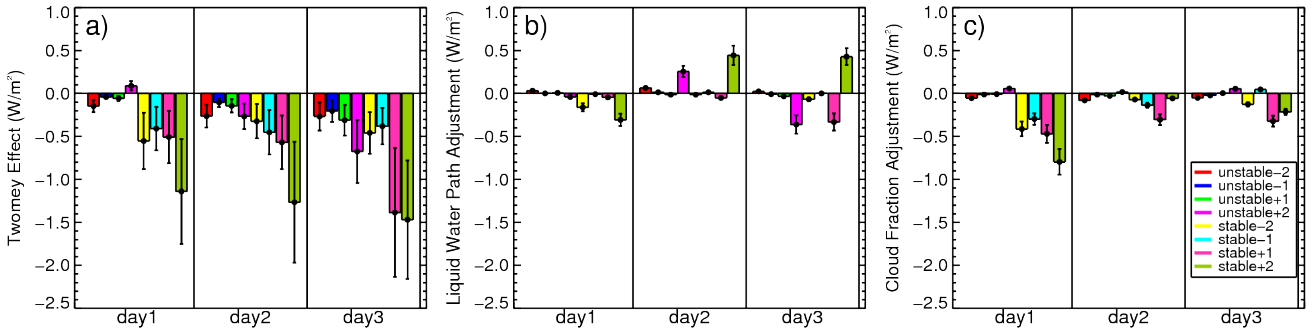

Fig. S13. Aerosol radiative effect for each meteorological stability regime extracted from the global-scale ensemble class of oceanic trajectories spanning 60° S – 60° N decomposed by the (a) Twomey effect, (b) liquid water path adjustment and (c) cloud fraction adjustment for daytime averages along trajectories. The 5 – 95th percentile confidence interval are given by the vertical bars.

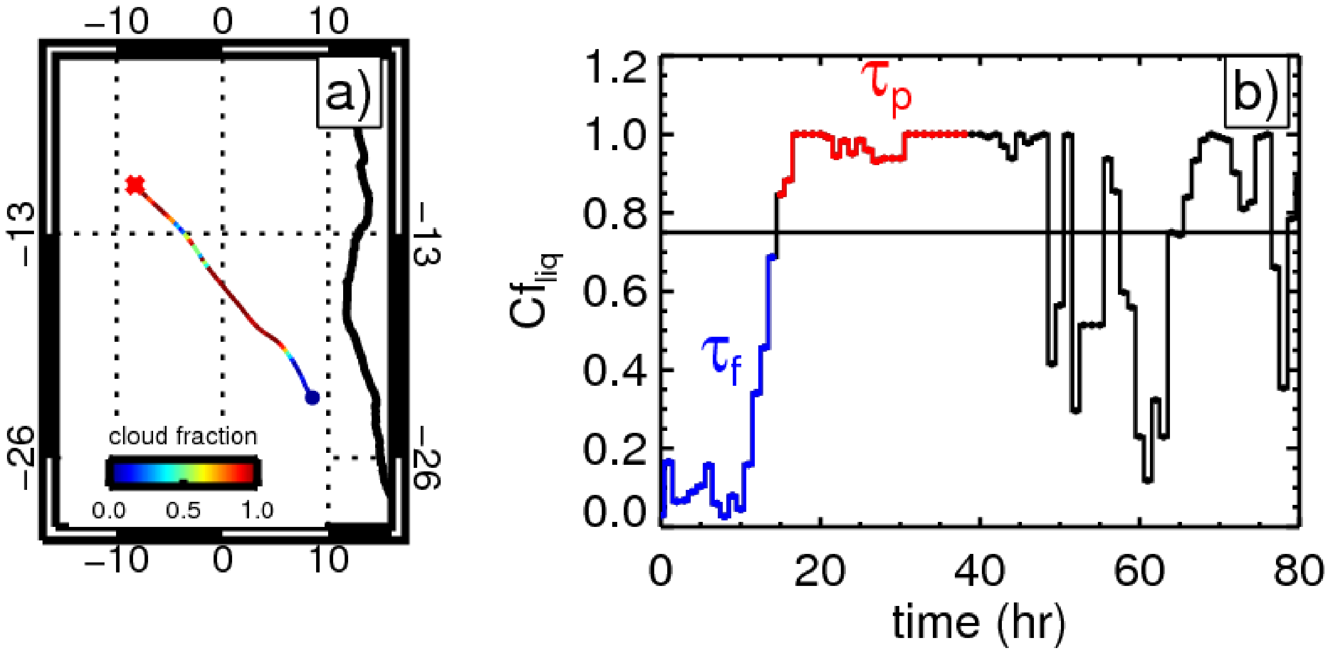

Fig. S14. Forward trajectory locations (a) initialized off the coast of Africa on February 3rd 2016 and plotted as a time-series (b) to depict cloud-formation timescale as defined by the amount of time it takes to increase cloud fraction from zero to over 0.75 (τ_f) and cloud-persistence timescale defined as the amount of time the cloud fraction remains above 0.75 for up to a maximum 24 hour period (τ_p).

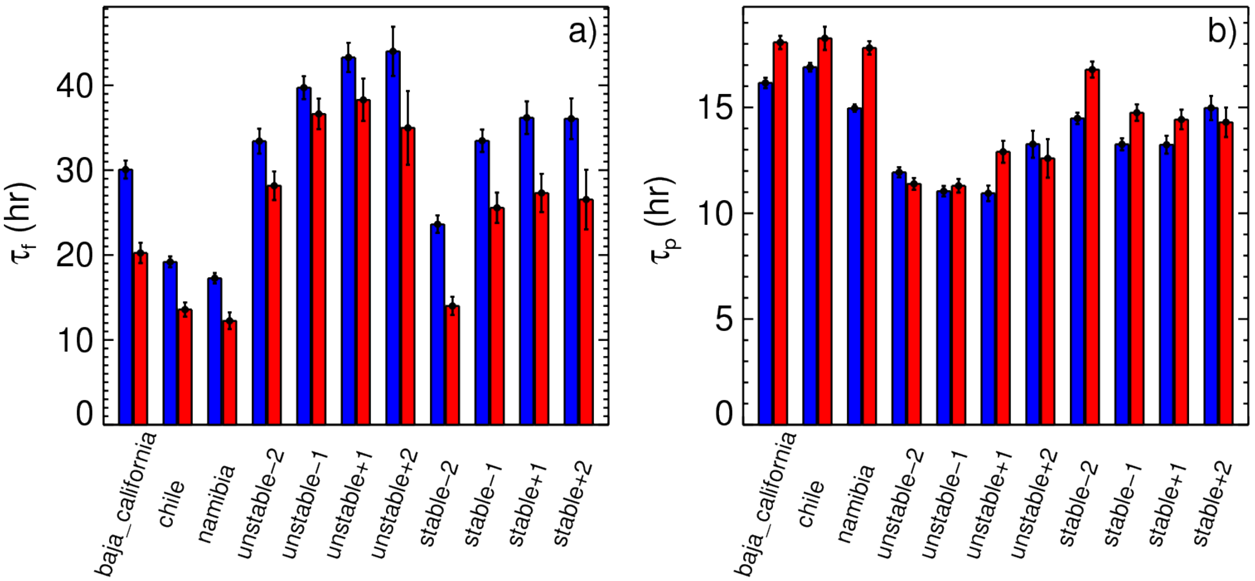

Fig. S15. Barchart of average timescale values for a) cloud formation and b) 24-hr persistence for the class of trajectories as observed by CERES SYN between 2015 – 2017 off the coasts of Baja California, Chile and Namibia and for the 8 composites of varying lower troposphere stability for clean and polluted trajectories as determined by an AOD threshold of 0.10. The 5 – 95th percentile confidence interval are given by the vertical bars.

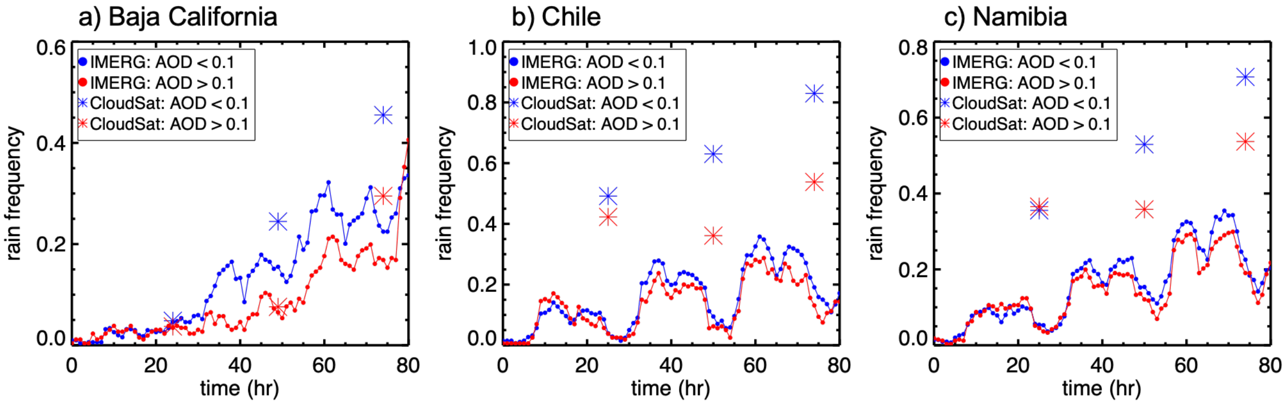

Fig. S16. Frequency of rain occurrence in trajectories located off the coasts of Baja California, Chile and Namibia using observations from the CloudSat cloud profiling radar and IMERG precipitation products for clean $(AOD < 0.1)$ and polluted $(AOD > 0.1)$ conditions.

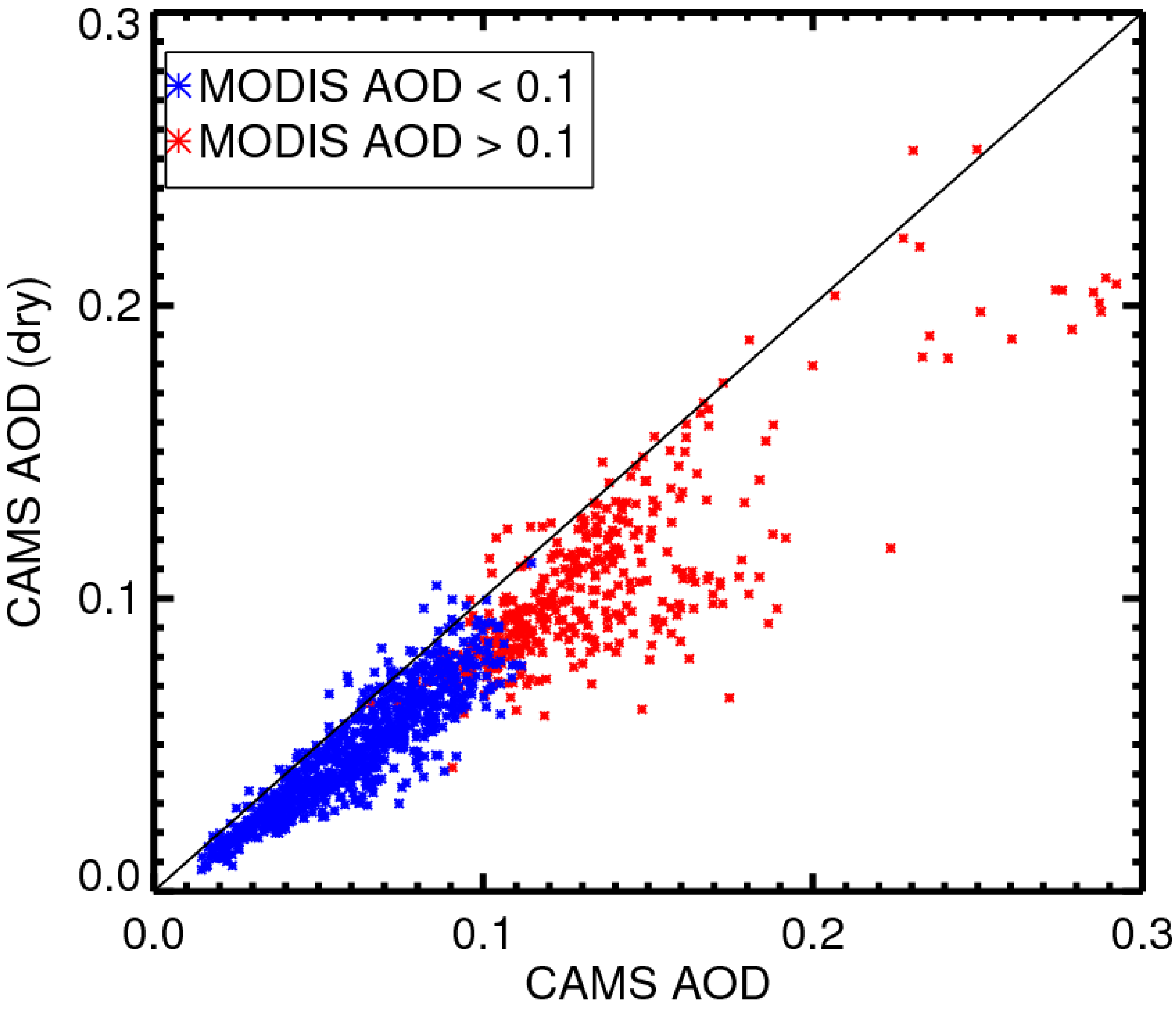

Fig. S17. Scatter plot of the dry AOD at a wavelength of 550 nm calculated from mass mixing ratios of 11 aerosol species from CAMS plotted against total AOD for the class of clean (blue) and polluted (red) trajectories sorted by MODIS retrieved AOD initialized off the coast of California

Movie S1. Animation of SEVIRI 0.64-μm reflectance image obtained on 5th September 2017 at 10:27 UTC overlain with the HYSPLIT back trajectories ending inside the Pocket of Open Cells (POC).
